# Syntheses and crystal structures of di­chlorido­(2,6-di­methyl­pyrazine-κ*N*)(methanol-κ*O*)zinc(II), di­bromido­(2,6-di­methyl­pyrazine-κ*N*)(methanol-κ*O*)zinc(II) and aqua­(2,6-di­methyl­pyrazine-κ*N*)di­iodido­zinc(II)

**DOI:** 10.1107/S2056989026002902

**Published:** 2026-03-24

**Authors:** Christian Näther

**Affiliations:** a Institut für Anorganische Chemie, Universität Kiel, Max-Eyth.-Str. 2, 24118 Kiel, Germany; University of Aberdeen, United Kingdom

**Keywords:** crystal structure, discrete complex, hydrogen bonding, synthesis, zinc halide, 2,6-di­methyl­pyrazine

## Abstract

Three new compounds with the compositions [ZnCl_2_(C_6_H_8_N_2_)(CH_3_OH)] (**1**), [ZnBr_2_(C_6_H_8_N_2_)(CH_3_OH)] (**2**) and [ZnI_2_(C_6_H_8_N_2_)(H_2_O)] (**3**) were prepared (C_6_H_8_N_2_ = 2,6-di­methyl­pyrazine). The asymmetric unit of each compound consists of one Zn^II^ cation, two halide anions, one 2,6-di­methyl­pyrazine ligand and one methanol (**1** and **2**) or water mol­ecule (**3**), with all atoms located in general positions. Compounds **1** and **2** are not isostructural. In the crystal structures, the metal cations are fourfold coordinated by two halide anions, one 2,6-di­methyl­pyrazine ligand and one methanol or water mol­ecule within a slightly distorted tetra­hedral geometry. In **1** and **2** the discrete complexes are linked into chains *via* O—H⋯O hydrogen bonds between the O—H H atom of the methanol mol­ecule and the 2,6-di­methyl­pyrazine N atom that is not involved in the metal coordination. These chains are further linked by weak C—H⋯Cl (**1**) or C—H⋯Br (**2**) inter­actions. In **3**, two complexes are linked by pairwise O—H⋯I hydrogen bonds into centrosymmetric dimeric units that are further connected by strong O—H⋯O hydrogen bonding between the second water H atom and the 2,6-di­methyl­pyrazine N-atom that is adjacent to the two methyl groups.

## Abstract 2

The syntheses and crystal structures of three complexes with the composition ZnCl_2_(2,6-di­methyl­pyrazine)(CH_3_OH) (**1**), ZnBr_2_(2,6-di­methyl­pyrazine)(CH_3_OH) (**2**) and ZnI_2_(2,6-di­methyl­pyrazine)(H_2_O) (**3**) are reported. Each consists of discrete complexes that are linked by hydrogen bonding.

## 1. Chemical context

Coordination compounds based on transition metal halides and N-donor coligands shows versatile structural behavior, which is especially the case for Cu^I^ halides (Kromp & Sheldrick, 1999 ▸; Peng *et al.*, 2010 ▸; Li *et al.*, 2005 ▸; Näther *et al.*, 2001 ▸, 2002 ▸). For one definite N-donor ligand and one definite halide anion, compounds of a different ratio between the metal halide and the coligand are observed in many cases (Näther & Jess, 2002 ▸).

In contrast, compounds based on twofold positively charged cations such as Zn^II^ show a limited structural behavior because in most cases only a tetra­hedral coordination is observed, leading to discrete complexes if monocoordinating coligands are used. However, there are a few examples for polymeric compounds, in which the Zn^II^ cations are in an octa­hedral coordination and linked into chains by μ-1,1-bridging halide anions (Pickardt & Staub, 1997 ▸; Saha *et al.*, 2017 ▸). Nevertheless, even for metals showing tetra­hedral coordination, compounds with a different stoichiometry and more condensed networks can be obtained if bridging instead of monocoordinating coligands such as, for example, pyrazine are used. With this ligand, compounds with the composition Zn*X*_2_(pyrazine)_2_ (*X* = Cl, Br) and Zn*X*_2_(pyrazine) (*X* = Cl, Br, I) have been reported (Bhosekar *et al.*, 2006 ▸; Bourne *et al.*, 200; Pickardt & Staub, 1997 ▸; Song *et al.*, 2004 ▸). In all of these compounds, the pyrazine ligand acts as bridging ligand.

In the course of our systematic investigations in this area, we became inter­ested in Zn*X*_2_ compounds based on 2,3-di­methyl­pyrazine. Because the methyl group is adjacent to the N atom, coordination of metal cations might be more difficult. In contrast to the pyrazine compounds, when ZnCl_2_ and 2,3-di­methyl­pyrazine were reacted, two compounds with the composition ZnCl_2_(2,3-di­methyl­pyrazine)_2_ and ZnCl_2_(2,3-di­methyl­pyrazine) were observed (Näther & Bhosekar, 2025*a* ▸). The 2,3-di­methyl­pyrazine-rich compound consists of discrete tetra­hedral complexes, in which the coligand is only terminally coordinated, whereas in the 2,3-di­methyl­pyrazine deficient compound the tetra­hedra are linked into chains by bridging 2,3-di­methyl­pyrazine ligands. The corresponding bromide compounds ZnBr_2_(2,3-di­methyl­pyrazine)_2_ (Yang *et al.*, 2025 ▸) and ZnBr_2_(2,3-di­methyl­pyrazine) (Näther & Bhosekar, 2025*b* ▸) have also been reported. With ZnI_2_, only the 2,3-di­methyl­pyrazine-deficient compound ZnI_2_(2,3-di­methyl­pyrazine was found, which forms discrete complexes and which is isotypic to ZnBr_2_(2,3-di­methyl­pyrazine) (Näther & Bhosekar, 2026 ▸).

Based on these results, we decided to prepare compounds with 2,6-di­methyl­pyrazine (C_6_H_8_N_2_), in which one of the N atoms is adjacent to both methyl groups, which make a metal coordination even more difficult. In this context, it is noted that one compound with the composition ZnI_2_(2,6-di­methyl­pyrazine)_2_ is already reported, which consists of discrete complexes in which the metal cations are coordinated by two iodide anions and two terminal 2,6-di­methyl­pyrazine ligands (Lee *et al.*, 2008 ▸). As expected, the 2,6-di­methyl­pyrazine ligand coordinates with the N atom that is not adjacent to the two methyl groups. As part of these investigations, we prepared and isolated crystals of the three title compounds, which were characterized by single crystal X-ray diffraction.
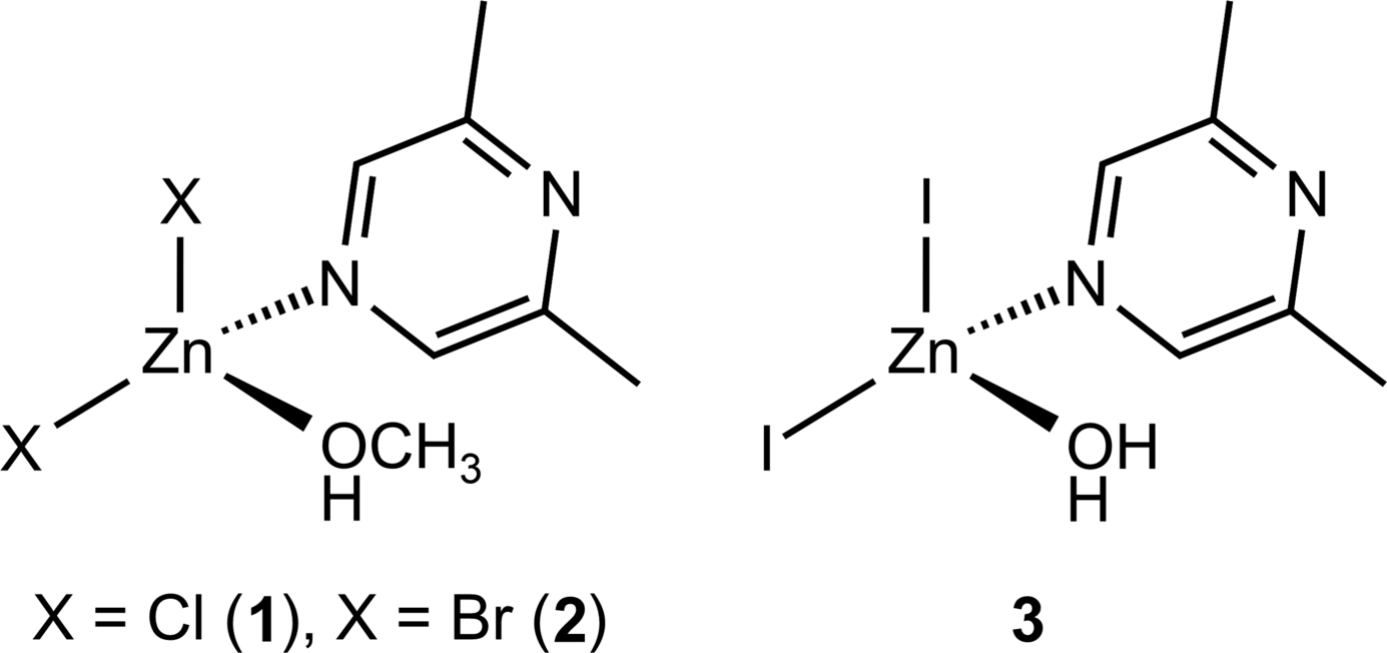


## 2. Structural commentary

**Figure 1:**
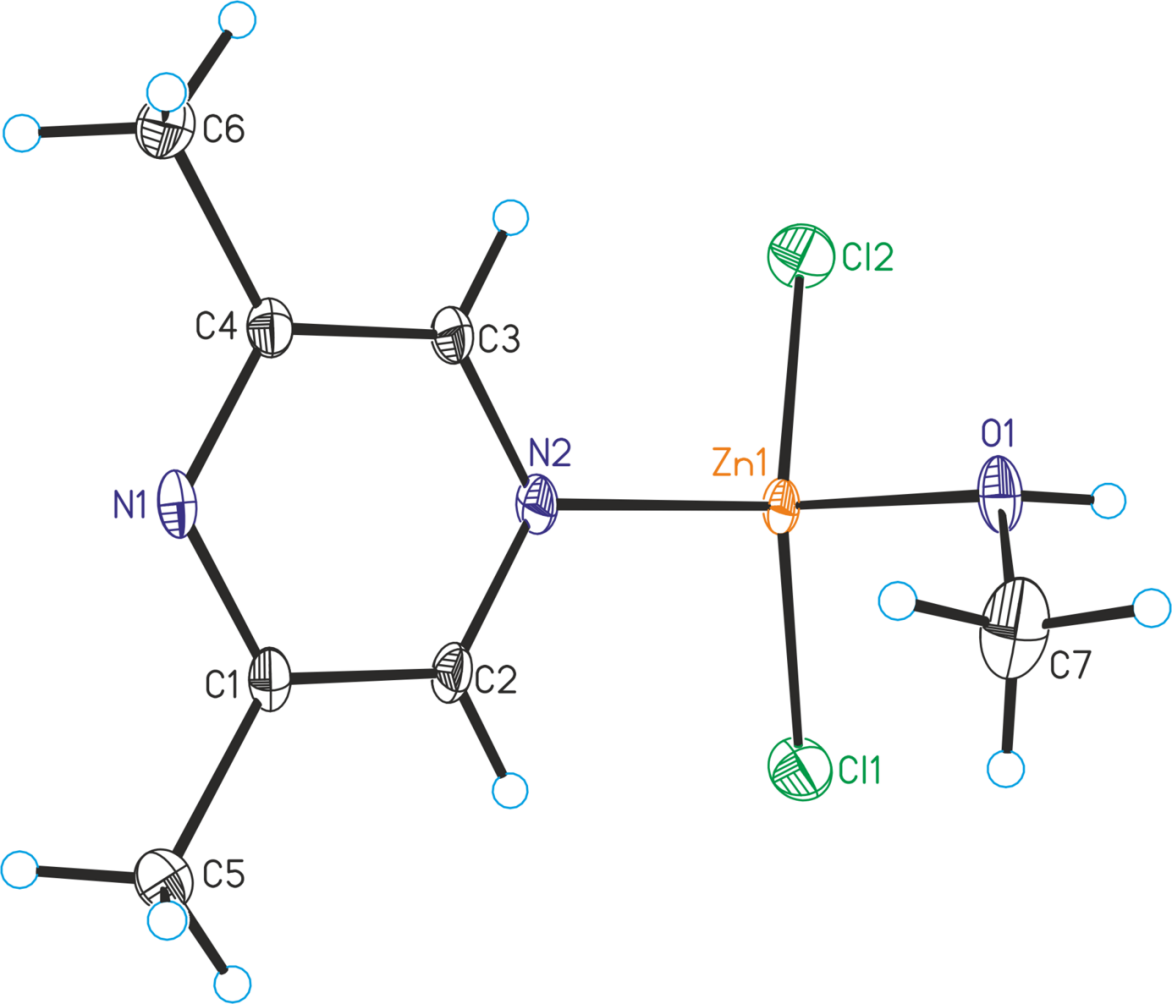
Crystal structures of **1** with labeling and displacement ellipsoids drawn at the 50% probability level.

The asymmetric units of ZnCl_2_(C_6_H_8_N_2_)(CH_3_OH) (**1**) and of ZnBr_2_(C_6_H_8_N_2_)(CH_3_OH) (**2**) consist of one Zn^II^ cation, two crystallographically independent halide anions, one 2,6-di­methyl­pyrazine ligand and one methanol mol­ecule, with all atoms lying on general crystallographic positions (Fig. 1 ▸). Compounds **1** (space group *P*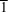) and **2** (space group *P*2_1_/*n*) are not isostructural.

In the crystal structures, the metal cations are tetra­hedrally coordinated by two halide anions, one methanol mol­ecule and one 2,6-di­methyl­pyrazine ligand that is coordinated by the N atom that is not adjacent to the methyl groups (Fig. 1 ▸). Bond lengths and angles shows that the tetra­hedra are strongly distorted with the halide–Zn–halide angles showing the largest values (Tables 1 ▸ and 2 ▸).

**Figure 2:**
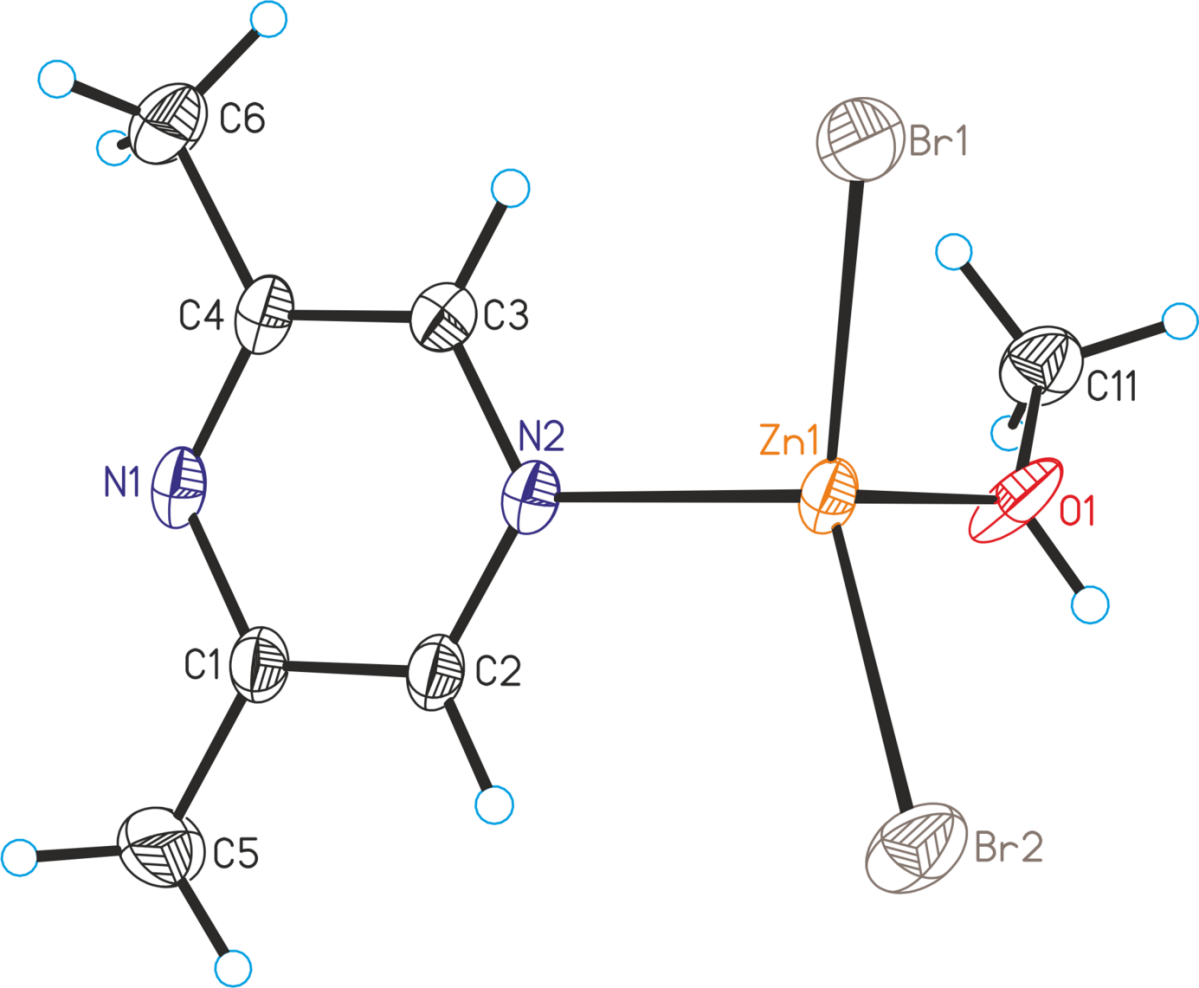
Crystal structures of **2** with labeling and displacement ellipsoids drawn at the 50% probability level.

The asymmetric unit of ZnI_2_(C_6_H_8_N_2_)(H_2_O) (**3**) consists of one Zn^II^ cation, two iodide anions, one 2,6-di­methyl­pyrazine ligand and one water mol­ecule in general positions (Fig. 2 ▸). The metal cations are fourfold coordinated by two halide anions, one 2,6-di­methyl­pyrazine ligand and one water mol­ecule (Fig. 1 ▸: bottom). As in compounds **1** and **2**, the coordination polyhedra can be described as strongly distorted tetra­hedra (Table 3 ▸).

As expected, in all three compounds the 2,6-di­methyl­pyrazine ligand is coordinated to the zinc cations with the N2 nitro­gen atom that is not adjacent to the two methyl groups because of steric crowding and this might also be the reason why no compounds with bridging 2,6-di­methyl­pyrazine ligands were isolated. This is in contrast to, for example, compounds with 2,3-di­methyl­pyrazine such as ZnCl_2_(2,3-di­methyl­pyrazine) (Näther & Bhosekar, 2025*a* ▸) and ZnBr_2_(2,3-di­methyl­pyrazine) (Näther & Bhosekar, 2025*b* ▸) in which the metal centers are linked by the 2,3-di­methyl­pyrazine ligands.

## 3. Supra­molecular features

**Figure 3:**
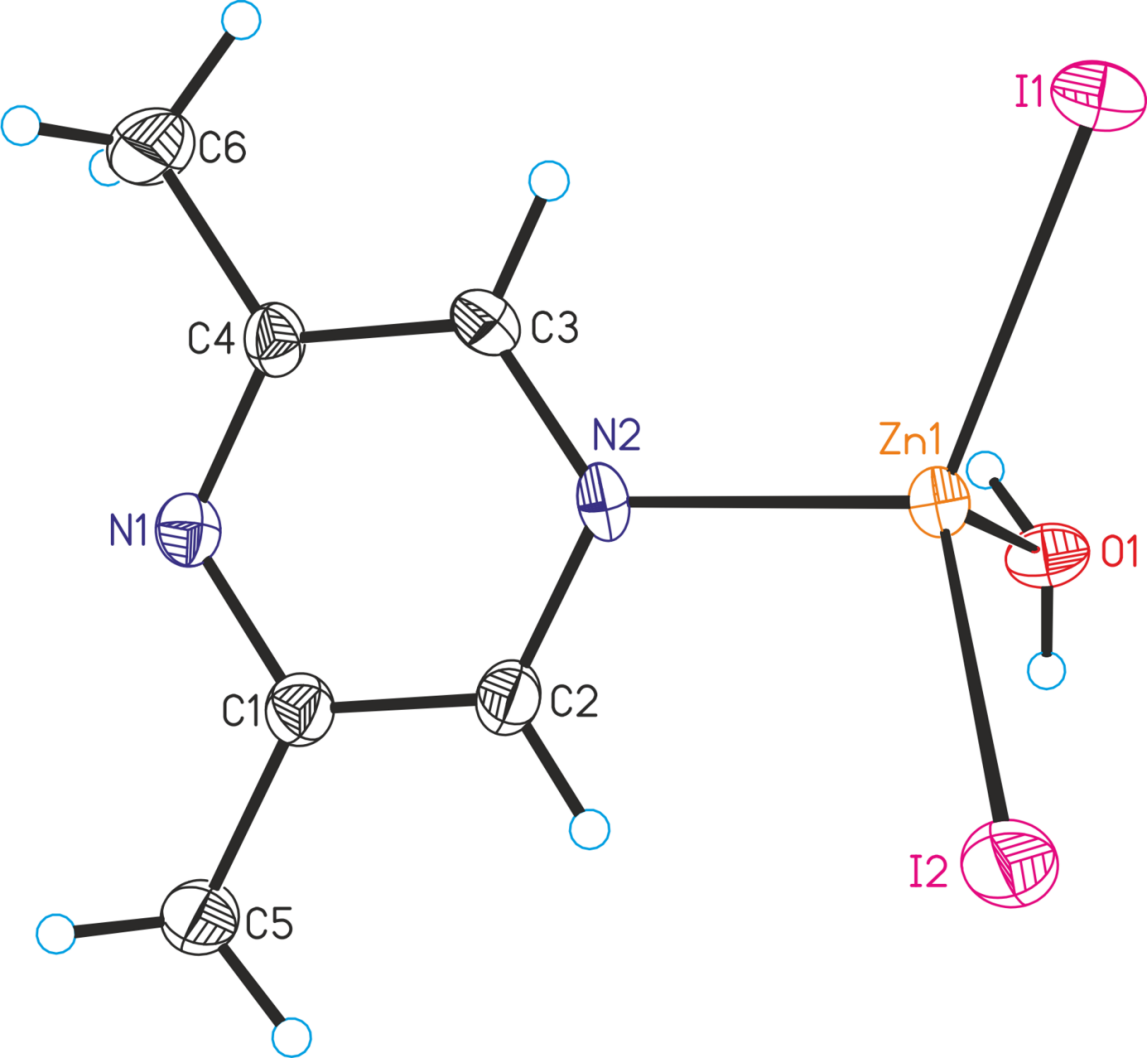
Crystal structure of **3** with labeling and displacement ellipsoids drawn at the 50% probability level.

In compound **1** and **2**, the discrete complexes are linked *via* O—H⋯N hydrogen bonds between the hydroxyl H atom of the methanol mol­ecule and the 2,6-di­methyl­pyrazine N atom that is not involved in the metal coordination (Fig. 3 ▸ and Tables 4 ▸ and 5 ▸). The O—H⋯N bond angles are close to linear and the H⋯N distances below 2 Å, indicating strong hydrogen bonds (Tables 4 ▸ and 5 ▸). The geometry of the chains in **1** and **2** is slightly different because of a different rotation of the methanol mol­ecule and the 2,6 di­methyl­pyrazine ligands.

**Figure 4:**
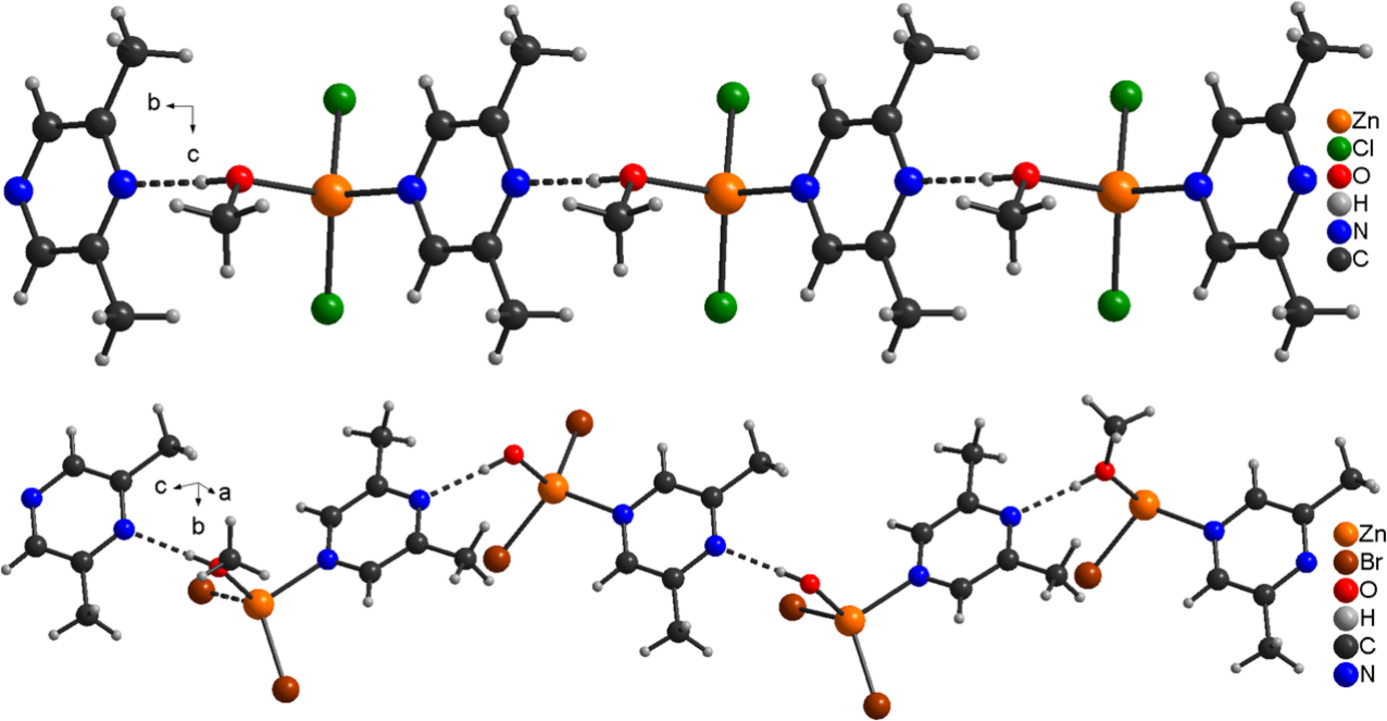
Crystal structure of **1** (top) and **2** (bottom) with view of a part of a chain. Inter­molecular O—H⋯N hydrogen bonds are shown as dashed lines.

These chains are inter­linked by a number of C—H⋯Cl and C—H⋯Br hydrogen bonds. The C—H⋯*X* angles (*X* = Cl, Br), especially for the chloride compound, are mostly close to linear, indicating stronger inter­actions (Figs. 4 ▸ and 5 ▸ and Tables 4 ▸ and 5 ▸).

**Figure 6:**
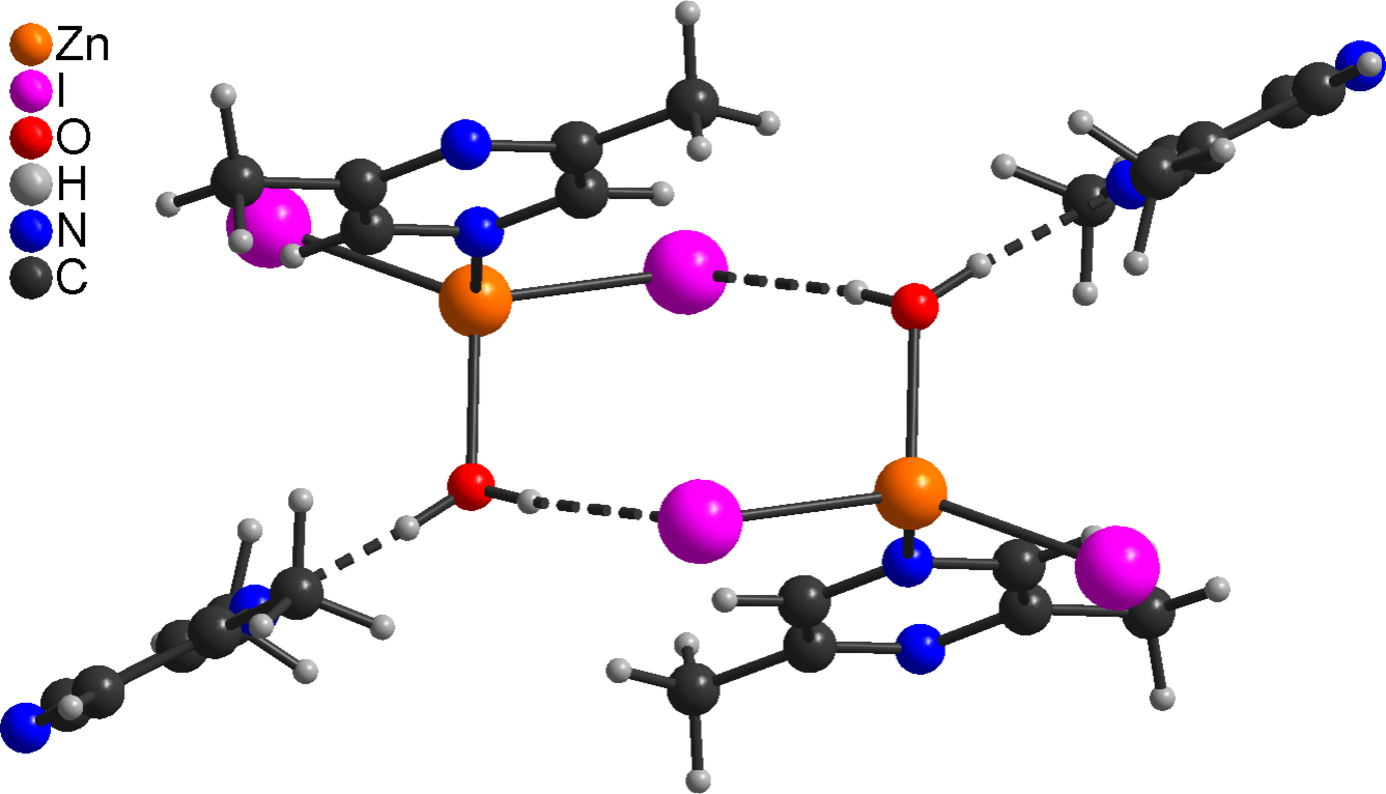
Crystal structure of **3** with view of a dimeric unit and C—H⋯I and O—H⋯N hydrogen bonds shown as dashed lines.

**Figure 7:**
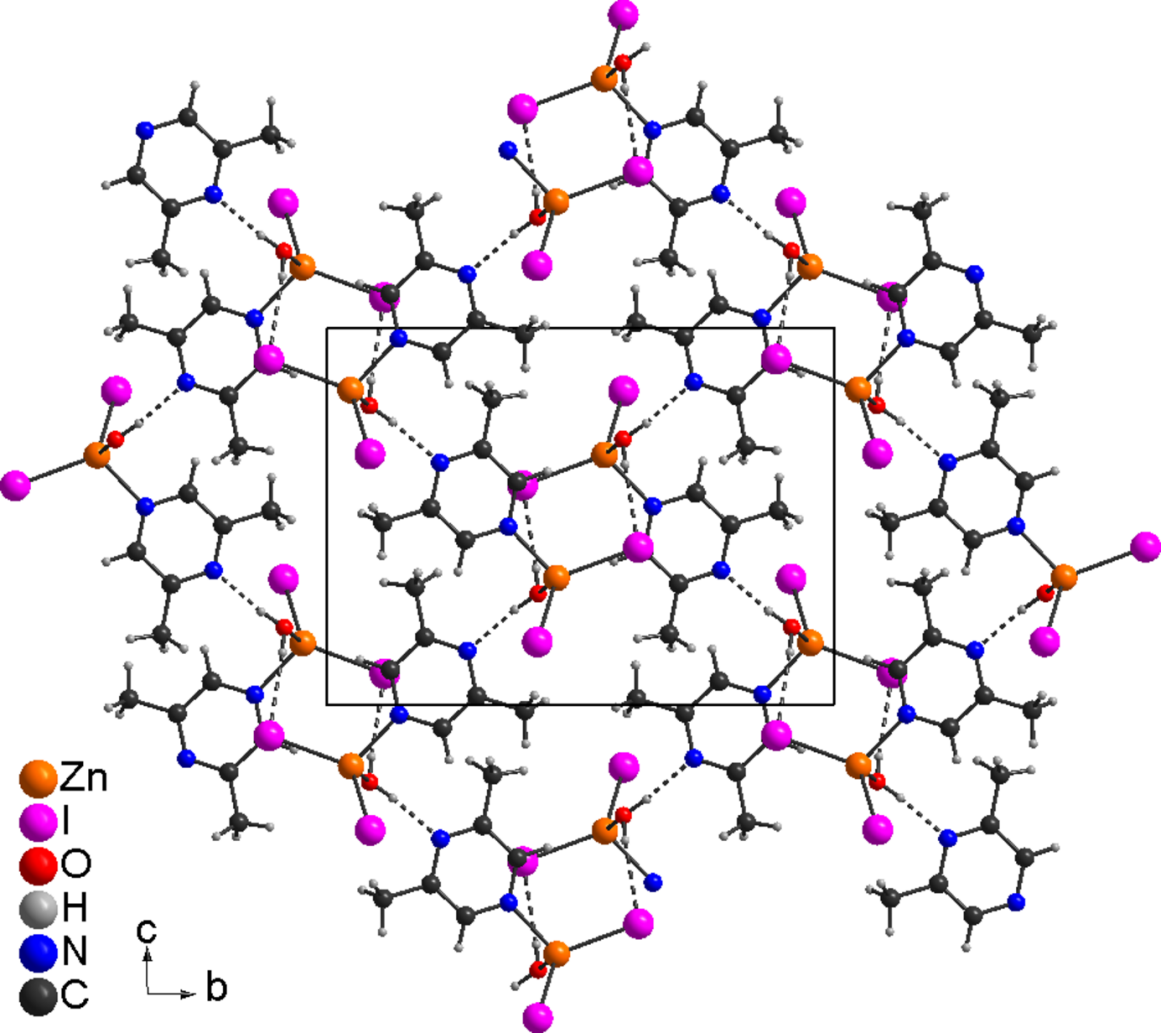
Crystal structure of **3** with view along the crystallographic *a*-axis direction and inter­molecular hydrogen bonding shown as dashed lines.

In compound **3**, two complexes are linked into dimers by centrosymmetric pairs of O—H⋯I hydrogen bonds between one of the water H atoms and the iodide anions, generating eight-membered rings. The O—H⋯I angle of 172 (5)° and the H⋯I distance of only 2.70 (2) Å indicate relatively strong hydrogen bonding (Fig. 6 ▸ and Table 6 ▸). These dimers are linked by strong O—H⋯N hydrogen bonds between the second water H atom and the 2,6-di­methyl­pyrazine ligands that are not involved in the metal coordination (Fig. 7 ▸ and Table 6 ▸). There are additional C—H⋯I inter­actions with much longer H⋯I distances indicating only weak inter­actions.

## 4. Database survey

A literature search revealed that only one coordination compound with Zn halides and 2,6-di­methyl­pyrazine is reported in the CSD (Version 5.43, 2025; Groom *et al.*, 2016 ▸) using CONQUEST (Bruno *et al.*, 2002 ▸). This is ZnI_2_(2,6-di­methyl­pyrazine)_2_ (CSD refcode XIYGIW; Lee *et al.*, 2008 ▸), in which the Zn cations are coordinated by two iodide anions and two terminal 2,6-di­methyl­pyrazine ligands into discrete complexes. Many more compounds are reported with pyrazine. These include ZnCl_2_(pyrazine)_2_ (REMPAB, Bhosekar *et al.*, 2006 ▸) and ZnBr_2_(pyrazine)_2_ (EBOLAI, Bourne *et al.*, 2001 ▸ and EBOLAI01, Bhosekar *et al.*, 2006 ▸) and Zn*X*_2_(pyrazine) [*X* = Cl (TISTAQ, Pickardt & Staub, 1996 ▸), Br (EBOKUB, Bourne *et al.*, 2001 ▸) and I (ISOPOV, Song *et al.*, 2004 ▸ and ISOPOV01, Bhosekar *et al.*, 2006 ▸)].

## 5. Synthesis and crystallization

**General**

Zinc chloride, zinc bromide and zinc iodide as well as 2,6-di­methyl­pyrazine were purchased from Sigma-Aldrich.

**Synthesis of 1**

0.500 mmol (68.1 mg) of zinc chloride and 1.00 mmol (108.1 mg 2,6-of di­methyl­pyrazine were reacted in 3 ml of methanol. Within 2 d, crystals were obtained suitable for single crystal X-ray diffraction.

**Synthesis of 2**

0.500 mmol (112.6 mg) of zinc bromide and 1.00 mmol (108.1 mg) of 2,6-di­methyl­pyrazine were reacted in 3 ml of methanol. Within 3 d, crystals were obtained suitable for single crystal X-ray diffraction.

**Synthesis of 3**

0.500 mmol (159.6 mg) of zinc iodide and 1.00 mmol (108.1 mg) of 2,6-di­methyl­pyrazine were reacted in 3 mL of a water/methanol mixture (1:1). Within 2 d, crystals were obtained suitable for single crystal X-ray diffraction.

## 6. Refinement

Crystal data, data collection and structure refinement details are summarized in Table 7 ▸. The C—H hydrogen atoms were positioned with idealized geometry (methyl H atoms allowed to rotate but not to tip) and were refined isotropically with *U*_iso_(H) = 1.2 *U*_eq_(C) (1.5 for methyl H atoms).

## Supplementary Material

Crystal structure: contains datablock(s) 1, 2, 3. DOI: 10.1107/S2056989026002902/hb8204sup1.cif

Structure factors: contains datablock(s) 1. DOI: 10.1107/S2056989026002902/hb82041sup2.hkl

Structure factors: contains datablock(s) 2. DOI: 10.1107/S2056989026002902/hb82042sup3.hkl

Structure factors: contains datablock(s) 3. DOI: 10.1107/S2056989026002902/hb82043sup4.hkl

CCDC references: 2539127, 2539126, 2539125

Additional supporting information:  crystallographic information; 3D view; checkCIF report

## Figures and Tables

**Figure 5:**
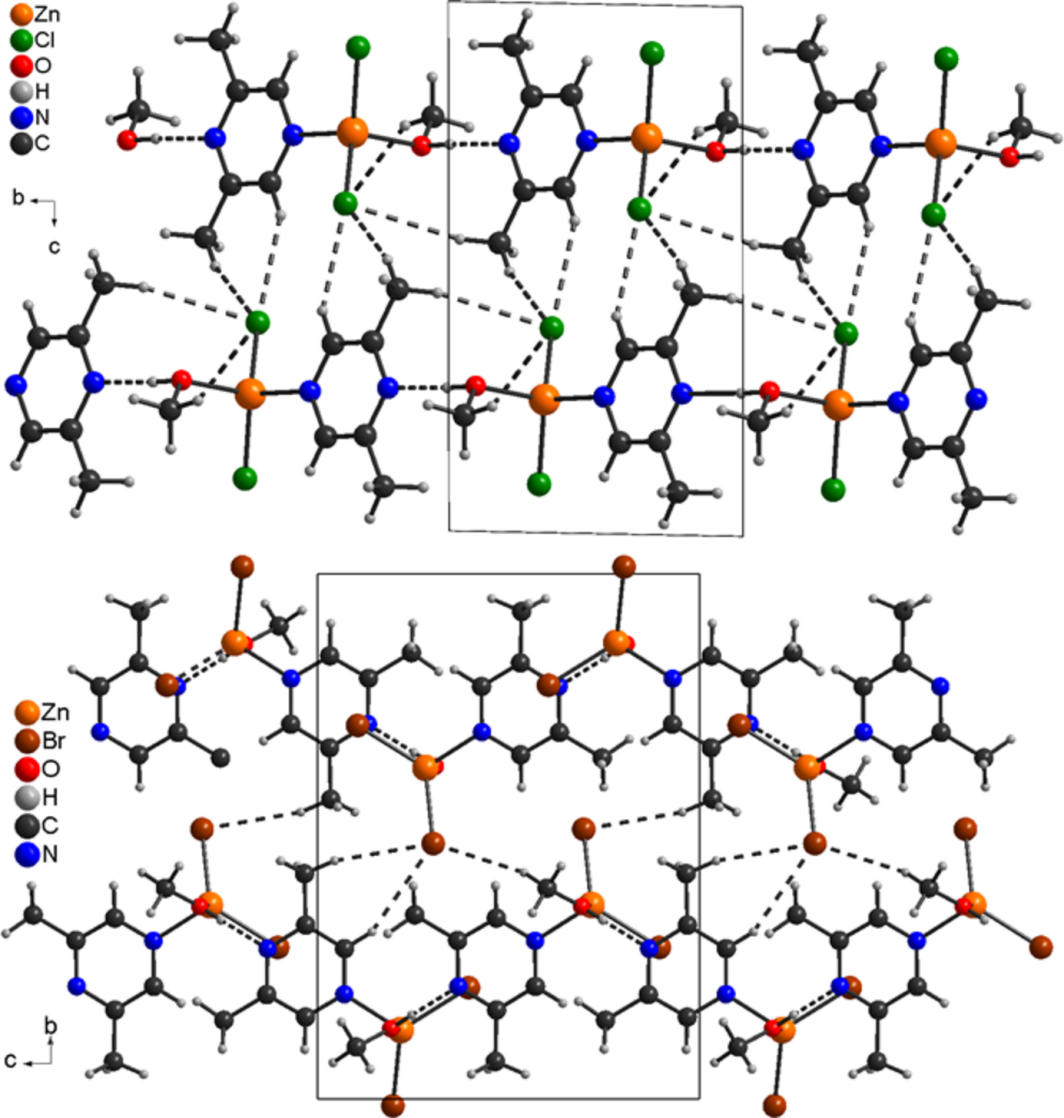
Crystal structure of **1** (top) and **2** (bottom) with view along the crystallographic *a*-axis direction and hydrogen bonds shown as dashed lines.

**Table 1:** Selected geometric parameters (Å, °) for **1**[Chem scheme1]

Zn1—Cl1	2.2088 (11)	Zn1—O1	2.024 (3)
Zn1—Cl2	2.2008 (10)	Zn1—N2	2.064 (3)
			
Cl2—Zn1—Cl1	122.64 (4)	O1—Zn1—N2	102.15 (11)
O1—Zn1—Cl1	106.68 (9)	N2—Zn1—Cl1	108.38 (9)
O1—Zn1—Cl2	104.91 (9)	N2—Zn1—Cl2	110.06 (9)

**Table 2:** Selected geometric parameters (Å, °) for **2**[Chem scheme1]

Zn1—Br1	2.3533 (5)	Zn1—O1	2.003 (2)
Zn1—Br2	2.3334 (5)	Zn1—N2	2.075 (3)
			
Br2—Zn1—Br1	123.16 (2)	O1—Zn1—N2	103.06 (11)
O1—Zn1—Br1	110.09 (9)	N2—Zn1—Br1	105.94 (7)
O1—Zn1—Br2	101.02 (7)	N2—Zn1—Br2	111.81 (8)

**Table 3:** Selected geometric parameters (Å, °) for **3**[Chem scheme1]

Zn1—I1	2.5557 (5)	Zn1—O1	2.022 (3)
Zn1—I2	2.5352 (5)	Zn1—N2	2.077 (3)
			
I2—Zn1—I1	121.276 (18)	O1—Zn1—N2	97.32 (12)
O1—Zn1—I1	103.93 (8)	N2—Zn1—I1	113.23 (9)
O1—Zn1—I2	112.16 (8)	N2—Zn1—I2	106.39 (9)

**Table 4:** Hydrogen-bond geometry (Å, °) for **1**[Chem scheme1]

*D*—H⋯*A*	*D*—H	H⋯*A*	*D*⋯*A*	*D*—H⋯*A*
O1—H1⋯N1^i^	0.84 (2)	1.90 (2)	2.738 (4)	173 (5)
C3—H3⋯Cl2^ii^	0.95	2.86	3.721 (4)	152
C5—H5*B*⋯Cl1^iii^	0.98	2.84	3.721 (4)	150
C5—H5*C*⋯Cl1^iv^	0.98	2.88	3.842 (4)	168
C6—H6*A*⋯Cl2^iv^	0.98	2.98	3.929 (4)	163
C6—H6*B*⋯Cl2^ii^	0.98	2.82	3.767 (4)	163
C7—H7*A*⋯Cl2^v^	0.98	2.98	3.876 (5)	153

Symmetry codes: (i) 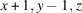; (ii) 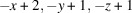; (iii) 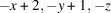; (iv) 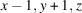; (v) 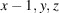.

**Table 5:** Hydrogen-bond geometry (Å, °) for **2**[Chem scheme1]

*D*—H⋯*A*	*D*—H	H⋯*A*	*D*⋯*A*	*D*—H⋯*A*
O1—H1⋯N1^i^	0.83 (2)	1.84 (2)	2.677 (3)	178 (5)
C11—H11*A*⋯Br2^ii^	0.98	3.13	3.767 (4)	124
C11—H11*C*⋯Br1^iii^	0.98	2.88	3.807 (4)	157
C2—H2⋯Br1^iv^	0.95	3.12	3.994 (3)	153
C3—H3⋯Br1	0.95	3.05	3.626 (3)	121
C5—H5*B*⋯Br1^iv^	0.98	2.95	3.903 (4)	166

Symmetry codes: (i) 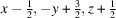; (ii) 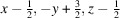; (iii) 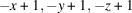; (iv) 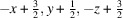.

**Table 6:** Hydrogen-bond geometry (Å, °) for **3**[Chem scheme1]

*D*—H⋯*A*	*D*—H	H⋯*A*	*D*⋯*A*	*D*—H⋯*A*
O1—H1*A*⋯I1^i^	0.86 (2)	2.70 (2)	3.555 (3)	172 (5)
O1—H1*B*⋯N1^ii^	0.85 (2)	1.87 (2)	2.708 (4)	172 (5)
C2—H2⋯I2	0.95	3.22	3.776 (4)	119
C5—H5*A*⋯I2^iii^	0.98	3.25	4.207 (5)	165

Symmetry codes: (i) 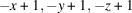; (ii) 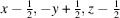; (iii) 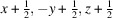.

**Table 7:** Experimental details

	**1**	**2**	**3**
Crystal data			
Chemical formula	[ZnCl_2_(C_6_H_8_N_2_)(CH_4_O)]	[ZnBr_2_(C_6_H_8_N_2_)(CH_4_O)]	[ZnI_2_(C_6_H_8_N_2_)(H_2_O)]
*M*_r_	276.46	365.38	445.33
Crystal system, space group	Triclinic, *P* 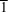	Monoclinic, *P*2_1_/*n*	Monoclinic, *P*2_1_/*n*
Temperature (K)	170	170	170
*a*, *b*, *c* (Å)	6.0481 (6), 7.4627 (7), 12.9967 (13)	7.1931 (5), 15.2428 (9), 11.4186 (6)	7.3914 (5), 14.7767 (8), 10.9917 (7)
α, β, γ (°)	89.945 (12), 85.471 (12), 74.710 (11)	90, 103.937 (7), 90	90, 94.883 (8), 90
*V* (Å^3^)	563.96 (10)	1215.11 (13)	1196.16 (13)
*Z*	2	4	4
Radiation type	Mo *K*α	Mo *K*α	Mo *K*α
μ (mm^−1^)	2.62	8.57	7.18
Crystal size (mm)	0.22 × 0.18 × 0.16	0.12 × 0.10 × 0.08	0.12 × 0.08 × 0.06

Data collection			
Diffractometer	Stoe IPDS2	Stoe IPDS2	Stoe IPDS2
Absorption correction	Numerical (*X-RED* and *X-SHAPE*; Stoe, 2008 ▸)	Numerical (*X-RED* and *X-SHAPE*; Stoe, 2008 ▸)	Numerical (*X-RED* and *X-SHAPE*; Stoe, 2008 ▸)
*T*_min_, *T*_max_	0.655, 0.765	0.236, 0.357	0.295, 0.510
No. of measured, independent and observed [*I* > 2σ(*I*)] reflections	5812, 2675, 2223	10027, 2943, 2434	12550, 2880, 2477
*R*_int_	0.048	0.031	0.040
(sin θ/λ)_max_ (Å^−1^)	0.660	0.662	0.661

Refinement			
*R*[*F*^2^ > 2σ(*F*^2^)], *wR*(*F*^2^), *S*	0.046, 0.132, 1.05	0.031, 0.078, 1.02	0.028, 0.070, 1.03
No. of reflections	2675	2943	2880
No. of parameters	125	125	118
No. of restraints	1	1	3
H-atom treatment	H atoms treated by a mixture of independent and constrained refinement	H atoms treated by a mixture of independent and constrained refinement	H atoms treated by a mixture of independent and constrained refinement
Δρ_max_, Δρ_min_ (e Å^−3^)	1.36, −1.32	0.47, −0.61	0.78, −0.93

Computer programs: *X-AREA* (Stoe, 2008 ▸), *SHELXT* (Sheldrick, 2015*a* ▸), *SHELXL* (Sheldrick, 2015*b* ▸), *DIAMOND* (Brandenburg, 1999 ▸), *XP* in *SHELXTL-PC* (Sheldrick, 2008 ▸) and *publCIF* (Westrip, 2010 ▸).

## supplementary crystallographic information

### Dichlorido(2,6-dimethylpyrazine-κN)(methanol-κO)zinc(II) (1) Crystal data

**Table:**

[ZnCl_2_(C_6_H_8_N_2_)(CH_4_O)]	*Z* = 2
*M**_r_* = 276.46	*F*(000) = 280
Triclinic, *P*1	*D*_x_ = 1.628 Mg m^−^^3^
*a* = 6.0481 (6) Å	Mo *K*α radiation, λ = 0.71073 Å
*b* = 7.4627 (7) Å	Cell parameters from 2716 reflections
*c* = 12.9967 (13) Å	θ = 2.9–28.1°
α = 89.945 (12)°	µ = 2.62 mm^−^^1^
β = 85.471 (12)°	*T* = 170 K
γ = 74.710 (11)°	Block, colorless
*V* = 563.96 (10) Å^3^	0.22 × 0.18 × 0.16 mm

### Dichlorido(2,6-dimethylpyrazine-κN)(methanol-κO)zinc(II) (1) Data collection

**Table:**

Stoe IPDS-2 diffractometer	2223 reflections with *I* > 2σ(*I*)
Graphite monochromator	*R*_int_ = 0.048
ω scans	θ_max_ = 28.0°, θ_min_ = 2.8°
Absorption correction: numerical (X-Red and X-Shape; Stoe, 2008)	*h* = −7→7
*T*_min_ = 0.655, *T*_max_ = 0.765	*k* = −9→9
5812 measured reflections	*l* = −17→17
2675 independent reflections	

### Dichlorido(2,6-dimethylpyrazine-κN)(methanol-κO)zinc(II) (1) Refinement

**Table:**

Refinement on *F*^2^	Hydrogen site location: mixed
Least-squares matrix: full	H atoms treated by a mixture of independent and constrained refinement
*R*[*F*^2^ > 2σ(*F*^2^)] = 0.046	*w* = 1/[σ^2^(*F*_o_^2^) + (0.0889*P*)^2^ + 0.0814*P*] where *P* = (*F*_o_^2^ + 2*F*_c_^2^)/3
*wR*(*F*^2^) = 0.132	(Δ/σ)_max_ = 0.001
*S* = 1.05	Δρ_max_ = 1.36 e Å^−^^3^
2675 reflections	Δρ_min_ = −1.32 e Å^−^^3^
125 parameters	Extinction correction: SHELXL-2016/6 (Sheldrick 2015b), Fc^*^=kFc[1+0.001xFc^2^λ^3^/sin(2θ)]^-1/4^
1 restraint	Extinction coefficient: 0.028 (5)
Primary atom site location: dual	

### Dichlorido(2,6-dimethylpyrazine-κN)(methanol-κO)zinc(II) (1) Special details

**Table:**

Geometry. All esds (except the esd in the dihedral angle between two l.s. planes) are estimated using the full covariance matrix. The cell esds are taken into account individually in the estimation of esds in distances, angles and torsion angles; correlations between esds in cell parameters are only used when they are defined by crystal symmetry. An approximate (isotropic) treatment of cell esds is used for estimating esds involving l.s. planes.

### Dichlorido(2,6-dimethylpyrazine-κN)(methanol-κO)zinc(II) (1) Fractional atomic coordinates and isotropic or equivalent isotropic displacement parameters (Å^2^)

**Table:**

	*x*	*y*	*z*	*U*_iso_*/*U*_eq_	
Zn1	1.02256 (6)	0.32540 (5)	0.25117 (3)	0.01229 (18)	
Cl1	1.18124 (16)	0.30675 (14)	0.09118 (8)	0.0246 (3)	
Cl2	1.21156 (16)	0.35079 (14)	0.38598 (8)	0.0241 (3)	
O1	0.9178 (4)	0.0914 (4)	0.2745 (2)	0.0172 (6)	
H1	1.028 (6)	−0.005 (5)	0.270 (4)	0.026*	
N1	0.2974 (5)	0.7935 (4)	0.2674 (2)	0.0124 (6)	
N2	0.7144 (5)	0.5283 (4)	0.2586 (2)	0.0133 (6)	
C1	0.3971 (6)	0.7158 (5)	0.1768 (3)	0.0132 (7)	
C2	0.6088 (6)	0.5818 (5)	0.1728 (3)	0.0139 (7)	
H2	0.678622	0.528008	0.108048	0.017*	
C3	0.6139 (6)	0.6067 (5)	0.3488 (3)	0.0140 (7)	
H3	0.687547	0.570667	0.410353	0.017*	
C4	0.4018 (6)	0.7410 (5)	0.3542 (3)	0.0133 (7)	
C5	0.2791 (7)	0.7769 (6)	0.0814 (3)	0.0215 (8)	
H5A	0.144983	0.726829	0.079890	0.032*	
H5B	0.385738	0.730979	0.020628	0.032*	
H5C	0.229148	0.912884	0.080948	0.032*	
C6	0.2844 (7)	0.8266 (6)	0.4547 (3)	0.0221 (8)	
H6A	0.237284	0.962092	0.448915	0.033*	
H6B	0.390538	0.792852	0.509089	0.033*	
H6C	0.148516	0.781027	0.472088	0.033*	
C7	0.7388 (7)	0.0526 (6)	0.2186 (4)	0.0287 (10)	
H7A	0.594142	0.147234	0.236440	0.043*	
H7B	0.720038	−0.070144	0.237140	0.043*	
H7C	0.780465	0.054397	0.144286	0.043*	

### Dichlorido(2,6-dimethylpyrazine-κN)(methanol-κO)zinc(II) (1) Atomic displacement parameters (Å^2^)

**Table:**

	*U*^11^	*U*^22^	*U*^33^	*U*^12^	*U*^13^	*U*^23^
Zn1	0.0077 (2)	0.0089 (2)	0.0179 (3)	0.00237 (15)	−0.00194 (15)	−0.00016 (15)
Cl1	0.0228 (5)	0.0263 (5)	0.0199 (5)	−0.0002 (4)	0.0051 (4)	0.0030 (4)
Cl2	0.0205 (5)	0.0272 (5)	0.0253 (5)	−0.0052 (4)	−0.0105 (4)	−0.0016 (4)
O1	0.0106 (12)	0.0105 (12)	0.0289 (15)	0.0007 (9)	−0.0032 (11)	0.0025 (11)
N1	0.0081 (13)	0.0080 (13)	0.0199 (16)	0.0000 (10)	−0.0013 (11)	0.0022 (11)
N2	0.0076 (13)	0.0114 (14)	0.0179 (15)	0.0028 (11)	−0.0016 (11)	0.0002 (11)
C1	0.0111 (15)	0.0083 (15)	0.0196 (18)	−0.0010 (12)	−0.0038 (13)	0.0000 (13)
C2	0.0110 (15)	0.0099 (16)	0.0181 (17)	0.0020 (12)	−0.0011 (13)	−0.0018 (13)
C3	0.0119 (15)	0.0114 (16)	0.0160 (17)	0.0024 (13)	−0.0030 (13)	0.0004 (13)
C4	0.0123 (16)	0.0104 (15)	0.0157 (17)	−0.0007 (12)	−0.0004 (13)	0.0012 (13)
C5	0.0200 (18)	0.0208 (19)	0.0194 (19)	0.0038 (15)	−0.0077 (15)	−0.0019 (15)
C6	0.0184 (18)	0.023 (2)	0.0178 (19)	0.0062 (15)	0.0004 (15)	−0.0009 (15)
C7	0.0178 (19)	0.020 (2)	0.049 (3)	−0.0050 (16)	−0.0101 (18)	−0.0034 (19)

### Dichlorido(2,6-dimethylpyrazine-κN)(methanol-κO)zinc(II) (1) Geometric parameters (Å, º)

**Table:**

Zn1—Cl1	2.2088 (11)	C3—H3	0.9500
Zn1—Cl2	2.2008 (10)	C3—C4	1.401 (5)
Zn1—O1	2.024 (3)	C4—C6	1.495 (5)
Zn1—N2	2.064 (3)	C5—H5A	0.9800
O1—H1	0.841 (19)	C5—H5B	0.9800
O1—C7	1.439 (5)	C5—H5C	0.9800
N1—C1	1.339 (5)	C6—H6A	0.9800
N1—C4	1.343 (5)	C6—H6B	0.9800
N2—C2	1.337 (5)	C6—H6C	0.9800
N2—C3	1.338 (5)	C7—H7A	0.9800
C1—C2	1.398 (4)	C7—H7B	0.9800
C1—C5	1.491 (5)	C7—H7C	0.9800
C2—H2	0.9500		
			
Cl2—Zn1—Cl1	122.64 (4)	N1—C4—C3	119.8 (3)
O1—Zn1—Cl1	106.68 (9)	N1—C4—C6	118.4 (3)
O1—Zn1—Cl2	104.91 (9)	C3—C4—C6	121.8 (3)
O1—Zn1—N2	102.15 (11)	C1—C5—H5A	109.5
N2—Zn1—Cl1	108.38 (9)	C1—C5—H5B	109.5
N2—Zn1—Cl2	110.06 (9)	C1—C5—H5C	109.5
Zn1—O1—H1	113 (4)	H5A—C5—H5B	109.5
C7—O1—Zn1	121.7 (2)	H5A—C5—H5C	109.5
C7—O1—H1	107 (3)	H5B—C5—H5C	109.5
C1—N1—C4	119.4 (3)	C4—C6—H6A	109.5
C2—N2—Zn1	120.4 (2)	C4—C6—H6B	109.5
C2—N2—C3	118.5 (3)	C4—C6—H6C	109.5
C3—N2—Zn1	121.1 (2)	H6A—C6—H6B	109.5
N1—C1—C2	120.1 (3)	H6A—C6—H6C	109.5
N1—C1—C5	118.6 (3)	H6B—C6—H6C	109.5
C2—C1—C5	121.3 (3)	O1—C7—H7A	109.5
N2—C2—C1	121.0 (3)	O1—C7—H7B	109.5
N2—C2—H2	119.5	O1—C7—H7C	109.5
C1—C2—H2	119.5	H7A—C7—H7B	109.5
N2—C3—H3	119.5	H7A—C7—H7C	109.5
N2—C3—C4	121.1 (3)	H7B—C7—H7C	109.5
C4—C3—H3	119.5		
			
Zn1—N2—C2—C1	178.4 (3)	C1—N1—C4—C6	−178.6 (3)
Zn1—N2—C3—C4	−178.3 (3)	C2—N2—C3—C4	0.5 (5)
N1—C1—C2—N2	0.4 (6)	C3—N2—C2—C1	−0.4 (5)
N2—C3—C4—N1	−0.4 (6)	C4—N1—C1—C2	−0.3 (5)
N2—C3—C4—C6	178.5 (4)	C4—N1—C1—C5	−179.6 (3)
C1—N1—C4—C3	0.4 (5)	C5—C1—C2—N2	179.6 (4)

### Dichlorido(2,6-dimethylpyrazine-κN)(methanol-κO)zinc(II) (1) Hydrogen-bond geometry (Å, º)

**Table:**

*D*—H···*A*	*D*—H	H···*A*	*D*···*A*	*D*—H···*A*
O1—H1···N1^i^	0.84 (2)	1.90 (2)	2.738 (4)	173 (5)
C3—H3···Cl2^ii^	0.95	2.86	3.721 (4)	152
C5—H5*B*···Cl1^iii^	0.98	2.84	3.721 (4)	150
C5—H5*C*···Cl1^iv^	0.98	2.88	3.842 (4)	168
C6—H6*A*···Cl2^iv^	0.98	2.98	3.929 (4)	163
C6—H6*B*···Cl2^ii^	0.98	2.82	3.767 (4)	163
C7—H7*A*···Cl2^v^	0.98	2.98	3.876 (5)	153

Symmetry codes: (i) *x*+1, *y*−1, *z*; (ii) −*x*+2, −*y*+1, −*z*+1; (iii) −*x*+2, −*y*+1, −*z*; (iv) *x*−1, *y*+1, *z*; (v) *x*−1, *y*, *z*.

### Dibromido(2,6-dimethylpyrazine-κN)(methanol-κO)zinc(II) (2) Crystal data

**Table:**

[ZnBr_2_(C_6_H_8_N_2_)(CH_4_O)]	*F*(000) = 704
*M**_r_* = 365.38	*D*_x_ = 1.997 Mg m^−^^3^
Monoclinic, *P*2_1_/*n*	Mo *K*α radiation, λ = 0.71073 Å
*a* = 7.1931 (5) Å	Cell parameters from 8000 reflections
*b* = 15.2428 (9) Å	θ = 7.2–27.3°
*c* = 11.4186 (6) Å	µ = 8.57 mm^−^^1^
β = 103.937 (7)°	*T* = 170 K
*V* = 1215.11 (13) Å^3^	Block, colorless
*Z* = 4	0.12 × 0.10 × 0.08 mm

### Dibromido(2,6-dimethylpyrazine-κN)(methanol-κO)zinc(II) (2) Data collection

**Table:**

Stoe IPDS-2 diffractometer	2434 reflections with *I* > 2σ(*I*)
Graphite monochromator	*R*_int_ = 0.031
ω scans	θ_max_ = 28.1°, θ_min_ = 2.3°
Absorption correction: numerical (X-Red and X-Shape; Stoe, 2008)	*h* = −9→9
*T*_min_ = 0.236, *T*_max_ = 0.357	*k* = −20→20
10027 measured reflections	*l* = −13→15
2943 independent reflections	

### Dibromido(2,6-dimethylpyrazine-κN)(methanol-κO)zinc(II) (2) Refinement

**Table:**

Refinement on *F*^2^	Hydrogen site location: mixed
Least-squares matrix: full	H atoms treated by a mixture of independent and constrained refinement
*R*[*F*^2^ > 2σ(*F*^2^)] = 0.031	*w* = 1/[σ^2^(*F*_o_^2^) + (0.0515*P*)^2^] where *P* = (*F*_o_^2^ + 2*F*_c_^2^)/3
*wR*(*F*^2^) = 0.078	(Δ/σ)_max_ = 0.001
*S* = 1.02	Δρ_max_ = 0.47 e Å^−^^3^
2943 reflections	Δρ_min_ = −0.61 e Å^−^^3^
125 parameters	Extinction correction: SHELXL-2016/6 (Sheldrick 2015b), Fc^*^=kFc[1+0.001xFc^2^λ^3^/sin(2θ)]^-1/4^
1 restraint	Extinction coefficient: 0.0084 (7)
Primary atom site location: dual	

### Dibromido(2,6-dimethylpyrazine-κN)(methanol-κO)zinc(II) (2) Special details

**Table:**

Geometry. All esds (except the esd in the dihedral angle between two l.s. planes) are estimated using the full covariance matrix. The cell esds are taken into account individually in the estimation of esds in distances, angles and torsion angles; correlations between esds in cell parameters are only used when they are defined by crystal symmetry. An approximate (isotropic) treatment of cell esds is used for estimating esds involving l.s. planes.

### Dibromido(2,6-dimethylpyrazine-κN)(methanol-κO)zinc(II) (2) Fractional atomic coordinates and isotropic or equivalent isotropic displacement parameters (Å^2^)

**Table:**

	*x*	*y*	*z*	*U*_iso_*/*U*_eq_	
Zn1	0.62772 (5)	0.63072 (2)	0.71740 (3)	0.01889 (11)	
Br1	0.73136 (5)	0.48630 (2)	0.69719 (3)	0.02990 (12)	
Br2	0.72234 (5)	0.71163 (3)	0.89530 (3)	0.03381 (12)	
O1	0.3423 (3)	0.6336 (2)	0.6914 (2)	0.0344 (6)	
H1	0.308 (7)	0.660 (3)	0.747 (4)	0.052*	
C11	0.1921 (5)	0.5986 (3)	0.6012 (3)	0.0309 (7)	
H11A	0.107236	0.646053	0.562825	0.046*	
H11B	0.119366	0.556534	0.637516	0.046*	
H11C	0.245122	0.568677	0.540544	0.046*	
N1	0.7234 (4)	0.78592 (19)	0.3686 (2)	0.0220 (5)	
N2	0.6734 (4)	0.69914 (17)	0.5698 (2)	0.0188 (5)	
C1	0.7187 (4)	0.8310 (2)	0.4688 (3)	0.0228 (6)	
C2	0.6960 (4)	0.7861 (2)	0.5705 (3)	0.0217 (6)	
H2	0.696644	0.817979	0.642087	0.026*	
C3	0.6748 (4)	0.6556 (2)	0.4683 (3)	0.0213 (6)	
H3	0.656328	0.593891	0.465830	0.026*	
C4	0.7024 (4)	0.6984 (2)	0.3666 (3)	0.0229 (6)	
C5	0.7385 (7)	0.9290 (2)	0.4656 (4)	0.0410 (9)	
H5A	0.861314	0.944069	0.447765	0.062*	
H5B	0.733555	0.953631	0.544044	0.062*	
H5C	0.633628	0.953363	0.402749	0.062*	
C6	0.7132 (6)	0.6496 (3)	0.2544 (3)	0.0341 (8)	
H6A	0.656530	0.685392	0.183474	0.051*	
H6B	0.642639	0.594258	0.250431	0.051*	
H6C	0.847488	0.637298	0.255916	0.051*	

### Dibromido(2,6-dimethylpyrazine-κN)(methanol-κO)zinc(II) (2) Atomic displacement parameters (Å^2^)

**Table:**

	*U*^11^	*U*^22^	*U*^33^	*U*^12^	*U*^13^	*U*^23^
Zn1	0.01907 (18)	0.02417 (19)	0.01512 (19)	−0.00124 (13)	0.00745 (13)	0.00053 (13)
Br1	0.0435 (2)	0.02313 (17)	0.02577 (19)	0.00434 (13)	0.01359 (15)	0.00339 (13)
Br2	0.02469 (18)	0.0514 (2)	0.02468 (19)	−0.00688 (15)	0.00469 (13)	−0.01538 (15)
O1	0.0184 (10)	0.0626 (18)	0.0240 (12)	−0.0024 (11)	0.0089 (9)	−0.0233 (12)
C11	0.0277 (16)	0.039 (2)	0.0248 (17)	−0.0036 (14)	0.0036 (13)	−0.0066 (15)
N1	0.0188 (12)	0.0312 (14)	0.0174 (13)	−0.0020 (10)	0.0072 (10)	0.0038 (11)
N2	0.0192 (12)	0.0229 (12)	0.0159 (12)	−0.0003 (10)	0.0070 (10)	0.0019 (10)
C1	0.0231 (15)	0.0269 (16)	0.0204 (15)	−0.0034 (12)	0.0092 (12)	0.0013 (12)
C2	0.0214 (14)	0.0276 (16)	0.0180 (15)	−0.0034 (12)	0.0090 (12)	0.0003 (12)
C3	0.0229 (14)	0.0224 (15)	0.0200 (15)	−0.0008 (11)	0.0077 (12)	−0.0015 (12)
C4	0.0206 (14)	0.0301 (16)	0.0204 (15)	−0.0008 (12)	0.0095 (12)	0.0012 (12)
C5	0.065 (3)	0.0275 (18)	0.034 (2)	−0.0106 (18)	0.0197 (19)	0.0004 (16)
C6	0.047 (2)	0.037 (2)	0.0227 (17)	−0.0013 (17)	0.0177 (16)	−0.0036 (15)

### Dibromido(2,6-dimethylpyrazine-κN)(methanol-κO)zinc(II) (2) Geometric parameters (Å, º)

**Table:**

Zn1—Br1	2.3533 (5)	C1—C2	1.391 (4)
Zn1—Br2	2.3334 (5)	C1—C5	1.503 (5)
Zn1—O1	2.003 (2)	C2—H2	0.9500
Zn1—N2	2.075 (3)	C3—H3	0.9500
O1—H1	0.833 (19)	C3—C4	1.387 (4)
O1—C11	1.406 (4)	C4—C6	1.499 (5)
C11—H11A	0.9800	C5—H5A	0.9800
C11—H11B	0.9800	C5—H5B	0.9800
C11—H11C	0.9800	C5—H5C	0.9800
N1—C1	1.342 (4)	C6—H6A	0.9800
N1—C4	1.343 (4)	C6—H6B	0.9800
N2—C2	1.335 (4)	C6—H6C	0.9800
N2—C3	1.337 (4)		
			
Br2—Zn1—Br1	123.16 (2)	N2—C2—C1	121.4 (3)
O1—Zn1—Br1	110.09 (9)	N2—C2—H2	119.3
O1—Zn1—Br2	101.02 (7)	C1—C2—H2	119.3
O1—Zn1—N2	103.06 (11)	N2—C3—H3	119.2
N2—Zn1—Br1	105.94 (7)	N2—C3—C4	121.7 (3)
N2—Zn1—Br2	111.81 (8)	C4—C3—H3	119.2
Zn1—O1—H1	112 (4)	N1—C4—C3	119.4 (3)
C11—O1—Zn1	132.8 (2)	N1—C4—C6	118.6 (3)
C11—O1—H1	115 (4)	C3—C4—C6	122.0 (3)
O1—C11—H11A	109.5	C1—C5—H5A	109.5
O1—C11—H11B	109.5	C1—C5—H5B	109.5
O1—C11—H11C	109.5	C1—C5—H5C	109.5
H11A—C11—H11B	109.5	H5A—C5—H5B	109.5
H11A—C11—H11C	109.5	H5A—C5—H5C	109.5
H11B—C11—H11C	109.5	H5B—C5—H5C	109.5
C1—N1—C4	119.8 (3)	C4—C6—H6A	109.5
C2—N2—Zn1	122.5 (2)	C4—C6—H6B	109.5
C2—N2—C3	118.1 (3)	C4—C6—H6C	109.5
C3—N2—Zn1	119.4 (2)	H6A—C6—H6B	109.5
N1—C1—C2	119.5 (3)	H6A—C6—H6C	109.5
N1—C1—C5	117.8 (3)	H6B—C6—H6C	109.5
C2—C1—C5	122.7 (3)		
			
Zn1—N2—C2—C1	−177.3 (2)	C1—N1—C4—C6	−178.5 (3)
Zn1—N2—C3—C4	179.2 (2)	C2—N2—C3—C4	1.2 (5)
N1—C1—C2—N2	−1.9 (5)	C3—N2—C2—C1	0.6 (5)
N2—C3—C4—N1	−1.8 (5)	C4—N1—C1—C2	1.3 (4)
N2—C3—C4—C6	177.1 (3)	C4—N1—C1—C5	−178.7 (3)
C1—N1—C4—C3	0.5 (4)	C5—C1—C2—N2	178.1 (3)

### Dibromido(2,6-dimethylpyrazine-κN)(methanol-κO)zinc(II) (2) Hydrogen-bond geometry (Å, º)

**Table:**

*D*—H···*A*	*D*—H	H···*A*	*D*···*A*	*D*—H···*A*
O1—H1···N1^i^	0.83 (2)	1.84 (2)	2.677 (3)	178 (5)
C11—H11*A*···Br2^ii^	0.98	3.13	3.767 (4)	124
C11—H11*C*···Br1^iii^	0.98	2.88	3.807 (4)	157
C2—H2···Br1^iv^	0.95	3.12	3.994 (3)	153
C3—H3···Br1	0.95	3.05	3.626 (3)	121
C5—H5*B*···Br1^iv^	0.98	2.95	3.903 (4)	166

Symmetry codes: (i) *x*−1/2, −*y*+3/2, *z*+1/2; (ii) *x*−1/2, −*y*+3/2, *z*−1/2; (iii) −*x*+1, −*y*+1, −*z*+1; (iv) −*x*+3/2, *y*+1/2, −*z*+3/2.

### Aqua(2,6-dimethylpyrazine-κN)diiodidozinc(II) (3) Crystal data

**Table:**

[ZnI_2_(C_6_H_8_N_2_)(H_2_O)]	*F*(000) = 816
*M**_r_* = 445.33	*D*_x_ = 2.473 Mg m^−^^3^
Monoclinic, *P*2_1_/*n*	Mo *K*α radiation, λ = 0.71073 Å
*a* = 7.3914 (5) Å	Cell parameters from 7998 reflections
*b* = 14.7767 (8) Å	θ = 8.5–27.1°
*c* = 10.9917 (7) Å	µ = 7.18 mm^−^^1^
β = 94.883 (8)°	*T* = 170 K
*V* = 1196.16 (13) Å^3^	Block, colorless
*Z* = 4	0.12 × 0.08 × 0.06 mm

### Aqua(2,6-dimethylpyrazine-κN)diiodidozinc(II) (3) Data collection

**Table:**

Stoe IPDS-2 diffractometer	2477 reflections with *I* > 2σ(*I*)
Graphite monochromator	*R*_int_ = 0.040
ω scans	θ_max_ = 28.0°, θ_min_ = 2.3°
Absorption correction: numerical (X-Red and X-Shape; Stoe, 2008)	*h* = −9→9
*T*_min_ = 0.295, *T*_max_ = 0.510	*k* = −19→19
12550 measured reflections	*l* = −14→14
2880 independent reflections	

### Aqua(2,6-dimethylpyrazine-κN)diiodidozinc(II) (3) Refinement

**Table:**

Refinement on *F*^2^	Hydrogen site location: mixed
Least-squares matrix: full	H atoms treated by a mixture of independent and constrained refinement
*R*[*F*^2^ > 2σ(*F*^2^)] = 0.028	*w* = 1/[σ^2^(*F*_o_^2^) + (0.0479*P*)^2^] where *P* = (*F*_o_^2^ + 2*F*_c_^2^)/3
*wR*(*F*^2^) = 0.070	(Δ/σ)_max_ < 0.001
*S* = 1.02	Δρ_max_ = 0.78 e Å^−^^3^
2880 reflections	Δρ_min_ = −0.93 e Å^−^^3^
118 parameters	Extinction correction: SHELXL-2016/6 (Sheldrick 2015b), Fc^*^=kFc[1+0.001xFc^2^λ^3^/sin(2θ)]^-1/4^
3 restraints	Extinction coefficient: 0.0043 (3)
Primary atom site location: dual	

### Aqua(2,6-dimethylpyrazine-κN)diiodidozinc(II) (3) Special details

**Table:**

Geometry. All esds (except the esd in the dihedral angle between two l.s. planes) are estimated using the full covariance matrix. The cell esds are taken into account individually in the estimation of esds in distances, angles and torsion angles; correlations between esds in cell parameters are only used when they are defined by crystal symmetry. An approximate (isotropic) treatment of cell esds is used for estimating esds involving l.s. planes.

### Aqua(2,6-dimethylpyrazine-κN)diiodidozinc(II) (3) Fractional atomic coordinates and isotropic or equivalent isotropic displacement parameters (Å^2^)

**Table:**

	*x*	*y*	*z*	*U*_iso_*/*U*_eq_	
Zn1	0.73068 (6)	0.45260 (3)	0.33652 (4)	0.01746 (11)	
I1	0.70914 (3)	0.61402 (2)	0.41751 (2)	0.02247 (9)	
I2	0.92934 (4)	0.41488 (2)	0.16648 (2)	0.03004 (10)	
O1	0.4691 (4)	0.41616 (19)	0.2948 (3)	0.0225 (6)	
H1A	0.417 (7)	0.412 (3)	0.361 (3)	0.034*	
H1B	0.433 (7)	0.371 (2)	0.252 (4)	0.034*	
N1	0.8726 (4)	0.2232 (2)	0.6430 (3)	0.0184 (6)	
N2	0.7913 (4)	0.3577 (2)	0.4732 (3)	0.0183 (6)	
C1	0.8679 (5)	0.2047 (3)	0.5226 (3)	0.0208 (7)	
C2	0.8277 (5)	0.2728 (2)	0.4380 (3)	0.0211 (7)	
H2	0.825714	0.259317	0.353358	0.025*	
C3	0.7957 (5)	0.3750 (2)	0.5925 (3)	0.0185 (7)	
H3	0.770384	0.434435	0.619252	0.022*	
C4	0.8369 (5)	0.3069 (2)	0.6790 (3)	0.0187 (7)	
C5	0.9078 (7)	0.1104 (3)	0.4843 (4)	0.0292 (9)	
H5A	1.024937	0.091071	0.524392	0.044*	
H5B	0.912702	0.108462	0.395508	0.044*	
H5C	0.811997	0.069732	0.507787	0.044*	
C6	0.8398 (8)	0.3260 (3)	0.8127 (4)	0.0356 (10)	
H6A	0.762378	0.282163	0.850627	0.053*	
H6B	0.794385	0.387313	0.824971	0.053*	
H6C	0.964498	0.320920	0.850212	0.053*	

### Aqua(2,6-dimethylpyrazine-κN)diiodidozinc(II) (3) Atomic displacement parameters (Å^2^)

**Table:**

	*U*^11^	*U*^22^	*U*^33^	*U*^12^	*U*^13^	*U*^23^
Zn1	0.0199 (2)	0.01571 (19)	0.0166 (2)	0.00201 (15)	0.00033 (16)	0.00198 (15)
I1	0.02262 (14)	0.01846 (13)	0.02691 (14)	−0.00292 (9)	0.00547 (10)	−0.00573 (9)
I2	0.03415 (17)	0.03106 (16)	0.02667 (15)	0.00725 (11)	0.01290 (11)	0.00196 (10)
O1	0.0219 (14)	0.0237 (13)	0.0218 (13)	−0.0040 (11)	0.0014 (11)	−0.0067 (11)
N1	0.0185 (15)	0.0168 (14)	0.0198 (15)	−0.0022 (12)	0.0012 (12)	0.0032 (11)
N2	0.0176 (15)	0.0172 (14)	0.0196 (14)	0.0044 (12)	−0.0009 (12)	0.0056 (12)
C1	0.0211 (18)	0.0201 (17)	0.0208 (17)	−0.0003 (14)	−0.0008 (14)	0.0002 (14)
C2	0.0241 (19)	0.0201 (17)	0.0183 (17)	0.0001 (14)	−0.0032 (15)	0.0011 (14)
C3	0.0184 (17)	0.0157 (15)	0.0217 (17)	0.0034 (13)	0.0036 (14)	−0.0006 (13)
C4	0.0197 (18)	0.0195 (17)	0.0170 (16)	0.0022 (14)	0.0029 (13)	0.0048 (13)
C5	0.043 (3)	0.0177 (17)	0.0255 (19)	0.0012 (17)	−0.0035 (18)	−0.0024 (15)
C6	0.059 (3)	0.032 (2)	0.0159 (19)	0.008 (2)	0.0074 (19)	0.0014 (16)

### Aqua(2,6-dimethylpyrazine-κN)diiodidozinc(II) (3) Geometric parameters (Å, º)

**Table:**

Zn1—I1	2.5557 (5)	C1—C5	1.492 (5)
Zn1—I2	2.5352 (5)	C2—H2	0.9500
Zn1—O1	2.022 (3)	C3—H3	0.9500
Zn1—N2	2.077 (3)	C3—C4	1.400 (5)
O1—H1A	0.858 (19)	C4—C6	1.495 (5)
O1—H1B	0.849 (19)	C5—H5A	0.9800
N1—C1	1.349 (5)	C5—H5B	0.9800
N1—C4	1.331 (5)	C5—H5C	0.9800
N2—C2	1.347 (5)	C6—H6A	0.9800
N2—C3	1.334 (5)	C6—H6B	0.9800
C1—C2	1.385 (5)	C6—H6C	0.9800
			
I2—Zn1—I1	121.276 (18)	N2—C3—H3	119.5
O1—Zn1—I1	103.93 (8)	N2—C3—C4	121.0 (3)
O1—Zn1—I2	112.16 (8)	C4—C3—H3	119.5
O1—Zn1—N2	97.32 (12)	N1—C4—C3	120.2 (3)
N2—Zn1—I1	113.23 (9)	N1—C4—C6	118.7 (3)
N2—Zn1—I2	106.39 (9)	C3—C4—C6	121.0 (3)
Zn1—O1—H1A	108 (3)	C1—C5—H5A	109.5
Zn1—O1—H1B	126 (4)	C1—C5—H5B	109.5
H1A—O1—H1B	106 (4)	C1—C5—H5C	109.5
C4—N1—C1	119.4 (3)	H5A—C5—H5B	109.5
C2—N2—Zn1	117.2 (2)	H5A—C5—H5C	109.5
C3—N2—Zn1	124.6 (2)	H5B—C5—H5C	109.5
C3—N2—C2	118.2 (3)	C4—C6—H6A	109.5
N1—C1—C2	119.8 (3)	C4—C6—H6B	109.5
N1—C1—C5	118.5 (3)	C4—C6—H6C	109.5
C2—C1—C5	121.6 (3)	H6A—C6—H6B	109.5
N2—C2—C1	121.3 (3)	H6A—C6—H6C	109.5
N2—C2—H2	119.3	H6B—C6—H6C	109.5
C1—C2—H2	119.3		
			
Zn1—N2—C2—C1	−179.4 (3)	C1—N1—C4—C6	179.0 (4)
Zn1—N2—C3—C4	179.7 (3)	C2—N2—C3—C4	0.0 (5)
N1—C1—C2—N2	−0.6 (6)	C3—N2—C2—C1	0.3 (6)
N2—C3—C4—N1	0.0 (6)	C4—N1—C1—C2	0.6 (6)
N2—C3—C4—C6	−179.3 (4)	C4—N1—C1—C5	−179.8 (4)
C1—N1—C4—C3	−0.3 (5)	C5—C1—C2—N2	179.8 (4)

### Aqua(2,6-dimethylpyrazine-κN)diiodidozinc(II) (3) Hydrogen-bond geometry (Å, º)

**Table:**

*D*—H···*A*	*D*—H	H···*A*	*D*···*A*	*D*—H···*A*
O1—H1*A*···I1^i^	0.86 (2)	2.70 (2)	3.555 (3)	172 (5)
O1—H1*B*···N1^ii^	0.85 (2)	1.87 (2)	2.708 (4)	172 (5)
C2—H2···I2	0.95	3.22	3.776 (4)	119
C5—H5*A*···I2^iii^	0.98	3.25	4.207 (5)	165

Symmetry codes: (i) −*x*+1, −*y*+1, −*z*+1; (ii) *x*−1/2, −*y*+1/2, *z*−1/2; (iii) *x*+1/2, −*y*+1/2, *z*+1/2.
